# Mathematics interest, self-efficacy, and anxiety predict STEM career choice in emerging adulthood

**DOI:** 10.1038/s41539-024-00275-1

**Published:** 2024-11-13

**Authors:** Rebecca Ferdinand, Margherita Malanchini, Kaili Rimfeld

**Affiliations:** 1 Department of Psychology, Royal Holloway, University of London, London, UK; 2 School of Biological and Behavioural Sciences, Queen Mary University of London, London, UK; 3 Social, Genetic and Developmental Psychiatry Centre, Institute of Psychiatry, Psychology and Neuroscience, King’s College London, London, UK

**Keywords:** Psychology, Education, Society, Human behaviour

## Abstract

To examine the combined effects of maths anxiety (MA), maths self-efficacy (MSE), and maths interest (MI) on STEM career choice, we analysed self-report data from 7908 Twins Early Development Study participants, collected at ages 16 (MSE, MI), 18 (MA) and 21 (STEM career choice). When analysed in the same model, MSE did not independently predict STEM career choice. MI (OR = 1.75) was a stronger predictor than MA (OR = 0.79), which was not significant after controlling for maths achievement. MI was a significant positive predictor of STEM career choices for both males (OR = 1.88) and females (OR = 1.77). However, MA was only predictive for males (OR = 0.62), and MSE was only predictive for females in the unadjusted model (OR = 1.77). These results highlight the importance of nurturing maths interest to bridge the STEM skills gap, regardless of sex. Future research should consider the co-development of maths-related psychological constructs to further understand their influence on STEM career paths.

## Introduction

The United Kingdom, like many nations^1–3^, is experiencing an ongoing shortage of people with Science, Technology, Engineering and Mathematics (STEM) expertise, with current estimates indicating a shortfall of over 173,000 workers across the sector^4^. A national inquiry has highlighted a need to address this skills gap to meet an expected increase in future demand and ensure sustainable economic growth^5^. Additionally, this issue may be worsened by the continued underrepresentation of women, with 27% of STEM workers being female, compared to 52% of the wider workforce^6^. Understanding why individuals may or may not choose to pursue a STEM career could be crucial in supporting the development of a globally competitive workforce.

While maths achievement in adolescence has long been demonstrated to predict STEM participation and career choice in adulthood^7,8^, research has shown that socio-cognitive and emotional factors may be as crucial for STEM career decision-making during this time^9,10^. Maths anxiety and maths motivational attitudes, such as maths self-efficacy and maths interest, are separately associated with student STEM participation^9,11–13^, although few studies have considered their combined impact on STEM career choice. Here, we tested the relationships between maths anxiety, maths self-efficacy, maths interest and STEM career participation in emerging adulthood using data from a UK-wide cohort.

Social cognitive career theory (SCCT) is a theory of motivation concerned with explaining the socio-cognitive processes behind career-related intentions and choices^14^. SCCT highlights self-efficacy and interest as key motivational attitudes influencing career-related behaviour^14,15^. In mathematics, self-efficacy refers to an individual’s perceived competence^16^, while interest is a motivational construct describing positive affect and orientation towards maths-related activities^17^. Both have been consistently linked to maths achievement^2,3,18^ and, in the case of maths self-efficacy, socioeconomic status^19,20^. In line with SCCT, studies confirm that maths self-efficacy and interest predict STEM participation. For example, a longitudinal study tracking 14,000 US students found that maths self-efficacy predicted enrolment intentions on STEM-related courses, independent of prior maths achievement and socioeconomic status^21^. Both maths self-efficacy and interest were also found to predict STEM course intentions in an Australian sample after controlling for maths achievement^13^. However, support for SCCT is not always consistent. Some studies report differential effects for each motivational construct. For example, one longitudinal study found a reciprocal relationship between maths interest and STEM career intentions over time but did not find a relationship between maths self-efficacy and career intentions^2^. Additionally, current research mainly focuses on career intentions rather than actual career choices and does not sufficiently address the emotional factors that could impact STEM-related behaviour.

Another aspect of mathematics linked to STEM career interest is maths anxiety. Maths motivational attitudes and maths anxiety are related^16,22^, yet distinct^23^. Maths anxiety, distinct from general anxiety, is characterised by negative emotional affect and tension at the prospect of, or during, mathematical tasks, and negatively correlates with maths achievement (average *r* = −0.30)^22–24^. Research also suggests that students from lower SES backgrounds are at increased risk of experiencing maths anxiety^24,25^. The interplay between maths anxiety and maths motivational attitudes is complex: while high maths anxiety often co-occurs with lower achievement, several studies have shown that motivational attitudes, including self-efficacy and interest, can buffer the adverse effects of maths anxiety on achievement^26,27^. For example, it has been demonstrated that, in individuals with low overall maths motivation, there is a negative relationship between maths anxiety and maths performance^27^. Conversely, in individuals with high maths motivation levels, this relationship becomes an inverted U, suggesting that a moderate level of maths anxiety might be performance-enhancing due to heightened alertness and focused attention before it becomes debilitating at higher levels^27,28^.

Research has also shown that, beyond the influence of previous maths achievement, students with high levels of maths anxiety are more likely to avoid STEM participation and are less inclined to pursue STEM-based careers^11,12,29,30^. Despite the existing literature on the non-linear joint effects of maths anxiety and maths motivational attitudes on maths achievement, few studies have considered how these factors might jointly influence STEM career choice. One study of high school students found that while maths anxiety did predict students’ self-perceptions of maths ability, maths performance expectancies and value perceptions, it did not predict STEM course enrolment intentions when accounting for these motivational constructs^31^. The study also found that, of these constructs, only value perceptions of maths predicted enrolment intentions^31^. This implies that the relationship between maths anxiety and STEM participation may be more complex than has been previously implied. It also suggests that different aspects of maths motivational attitudes may vary in their influence on STEM participation. However, as only course intentions were considered as an outcome, rather than actual STEM participation behaviour, this study’s relevance to understanding why individuals pursue STEM careers in adulthood may be limited. By investigating the joint influence of maths anxiety, maths self-efficacy and maths interest on individuals’ STEM career choices in emerging adulthood, the current investigation aims to address a gap in current research. Sex differences in maths anxiety, maths self-efficacy, maths interest and STEM participation have been consistently demonstrated across adolescence and emerging adulthood. Male participants tend to report higher maths self-efficacy and interest levels than female participants^9,13,32–34^. Conversely, female participants tend to report higher levels of maths anxiety compared to male participants^11,12,16,30^. They are also less likely to study STEM subjects in higher education^12,35–37^ and commit to STEM-based careers^35^ despite exhibiting similar levels of previous maths achievement^33,38^. Research exploring the influence of maths motivational attitudes on STEM participation and career choice reports very few sex differences in the relationship^21,35,39^. However, one study noted that maths self-efficacy was associated with STEM career interest in females but not males, whereas maths interest was associated for males but not females^13^. Fewer studies have considered sex differences in the impact of maths anxiety on STEM career choice, however, longitudinal research has shown that, in males, but not females, higher maths anxiety predicts lower future maths self-efficacy^37^. This finding may indicate that, like maths self-efficacy, the STEM career choices of males may be more negatively influenced by previous maths anxiety than those of females.

To mitigate the STEM skills gap in the UK, it is essential to understand the psychological factors shaping career choices in this sector. Our study employs longitudinal data from a large, nationally representative cohort to examine how maths anxiety, maths self-efficacy and maths interest affect STEM career selection in early adulthood. In this investigation, we aim to address the following questions: (1) What is the joint influence of maths anxiety (with and without controlling for general anxiety), maths self-efficacy and maths interest on STEM career choice in emerging adulthood? (2) Are there sex differences in the joint influence of maths anxiety, maths self-efficacy and maths interest on STEM career choice? Informed by SCCT and previous research work, we expected that maths self-efficacy and maths interest would each positively and independently predict STEM career choice. We also expected that maths anxiety would independently and negatively predict STEM career participation. Given the established associations between these factors and maths achievement, as well as socioeconomic status, we expected that these effects would be partially attenuated when controlling for these variables. Based on prior work considering sex differences, we expected that maths anxiety and maths interest would have stronger associations with STEM career choice in the male cohort than the female cohort and that maths self-efficacy would have a stronger association with STEM career choice in females than males. Our research plan was preregistered in the Open Science Framework (OSF; https://osf.io/y36bg).

## Results

### Descriptive statistics and mean-level sex differences

The descriptive statistics for continuous variables are presented in Supplementary Table 1. Frequency count and proportion statistics for STEM career choice, a binary variable, are presented in Supplementary Table 2. 2254 participants reported a degree and/or apprenticeship choice. 5.1% (117) reported an apprenticeship choice and 29.4% (662) reported a STEM career choice. Participants who reported a STEM career choice had a significantly higher mean GCSE mathematics achievement (M = 9.5, SD = 1.17) than the whole sample (M = 8.9, SE = 1.44; t(4599) = −24.74, *p* < 0.001).

Univariate ANOVAs were used to test for mean-level sex differences across all continuous variables, as shown in Supplementary Table 3. Small but significant differences emerged, with sex explaining a small to moderate proportion of the variance across measures (η² = 0.001–0.06). Females had significantly higher levels of maths anxiety (F(948, 541) = 86.68, *p* < 0.001, η² = 0.06) and general anxiety (F(948, 541) = 57.74, *p* < 0.001, η² = 0.04), while males showed significantly higher levels of maths self-efficacy (F(1485, 1074) = 149.10, *p* < 0.001, η² = 0.05) and maths interest (F(1485, 1074) = 39.04, *p* < 0.001, η² = 0.01). Although males also had significantly higher maths GCSE achievement and SES levels, sex explained 0.1% and 0.007% of the variance, respectively. All variables were therefore adjusted for sex and age using a regression method. The standardised residuals corrected for age and sex were used for all downstream analyses.

Supplementary Table 4 presents contingencies for sex differences in the STEM career choice. 63.9% of the sample were female. 21.9% of female participants chose a STEM career, and 42.5% of male participants chose a STEM career. A Pearson’s Chi-square test and an odds ratio calculation were performed to determine whether the proportion of STEM career choice differed between males and females and the effect size of this difference. The proportion of STEM career choices was found to differ significantly by sex, with males being more than 2.5 times as likely to make a STEM career choice than females, (X^2^(1, *N* = 2254) = 105, *p* < 0.001, OR = 2.63).

### The joint influence of maths anxiety, maths self-efficacy, and maths interest on STEM career choice in emerging adulthood

**Fig. 1:**
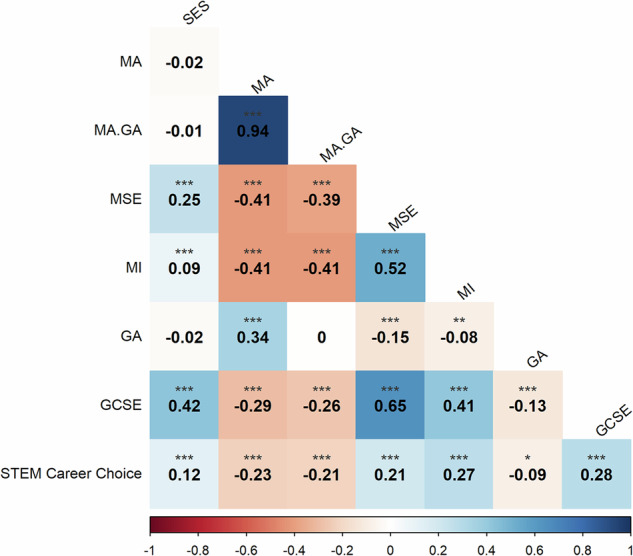
Correlations between all variables. Zero-order correlations between all variables. MA maths anxiety, MA.GA maths anxiety (independent of general anxiety), MSE maths self-efficacy, MI maths interest, GA general anxiety, GCSE GCSE maths achievement, SES socioeconomic status. *p < 0.05, **p < 0.01, ***p < 0.001.

Figure 1 presents Pearson’s correlations between all variables. Supplementary Table 5 reports all correlations with sample sizes and 95% confidence intervals. A negative correlation was observed between maths anxiety and STEM career choice (*r* = −0.23). A small positive correlation was observed between maths motivation and STEM career choice (*r* = 0.28). Small associations were observed between all other variables and STEM career choice (*r* ranging between −0.09 and 0.28). Maths anxiety had a small negative association with maths achievement (*r* = −0.29) and was moderately correlated to the same extent with both maths self-efficacy and maths interest (*r* = −0.41). Maths achievement was highly correlated with maths self-efficacy (*r* = 0.65) and moderately associated with maths interest (*r* = 0.41).

There was a moderate positive correlation between maths anxiety and general anxiety (*r* = 0.34). The association between general anxiety and STEM career choice was weak (*r* = −0.09), and there was no significant difference observed between the correlations for maths anxiety and STEM career choice (*r* = −0.23, 95% CI [−0.29, −0.17]) and maths anxiety (independent of general anxiety) and STEM career choice (*r* = −0.21, 95% CI [−0.27, −0.15]). We, therefore, used the maths anxiety variable uncorrected for general anxiety for the regression models.

We conducted three binary logistic regression models to assess the unique contributions of maths anxiety and maths motivation to STEM career choice. In the first model, maths anxiety, maths self-efficacy, and maths interest were jointly assessed. In the second model, maths anxiety, maths self-efficacy and maths interest were adjusted for GCSE maths achievement. In the third model, maths anxiety, maths self-efficacy and maths interest were adjusted for maths achievement and SES. Variance inflation factors (VIF) for each predictor variable in each model were all less than 1.6, meeting the logistic regression assumption of low or no multicollinearity between predictors (see Supplementary Table 6 for full VIF for each predictor variable).

**Fig. 2:**
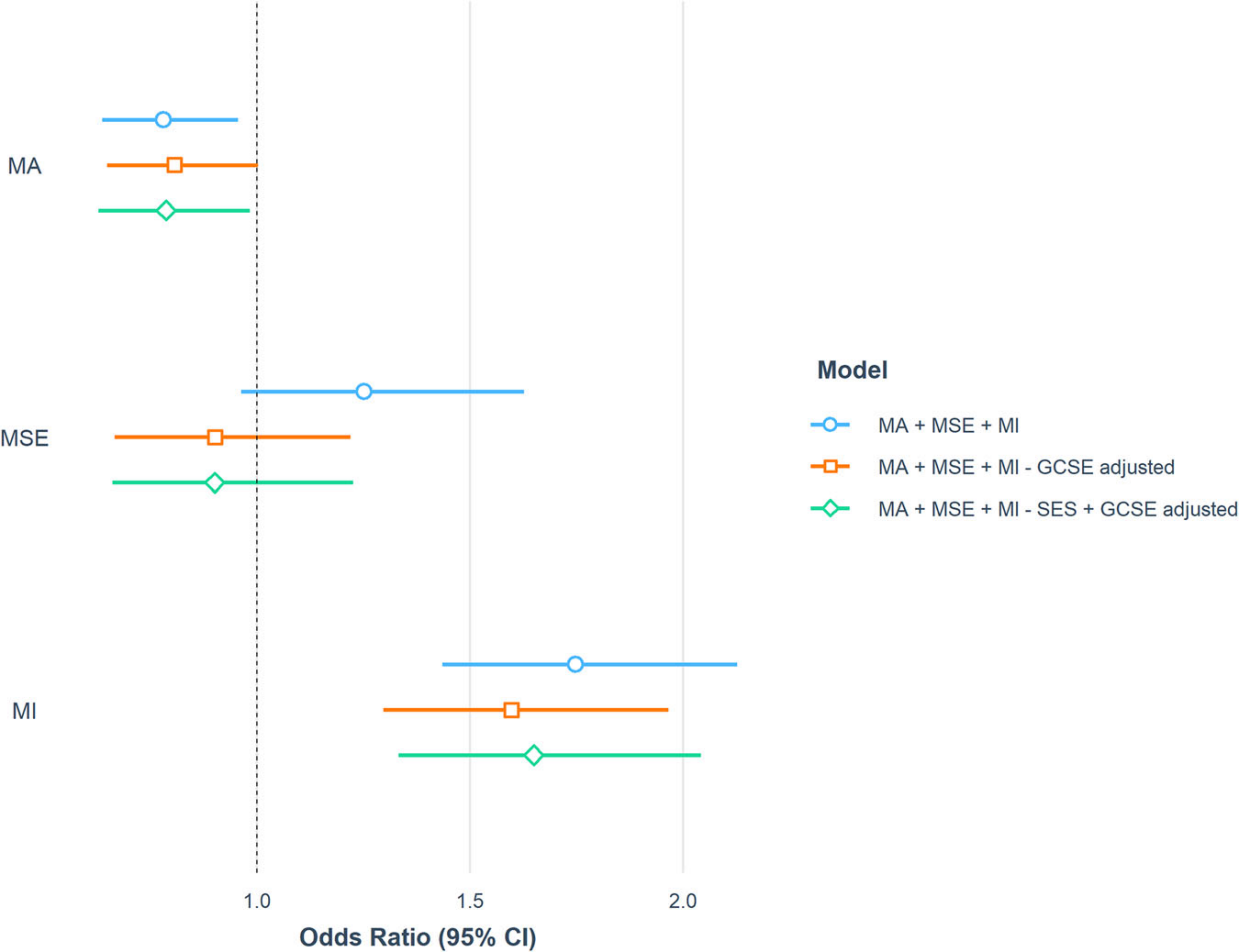
Joint associations between maths anxiety, maths self-efficacy and maths interest and STEM career choice. Relative ORs and 95% CIs of maths anxiety, maths self-efficacy and maths interest on STEM career choice across three binary logistic regression models. MA maths anxiety, MSE maths self-efficacy, MI maths interest, GCSE GCSE maths achievement, SES socioeconomic status.

The Nagelkerke pseudo-R-squared values for all models ranged between 0.15 and 0.21. Supplementary Table 7 summarises the findings of each regression model. Figure 2 visualises the relative effects of maths anxiety and maths motivation across all three models. Results from the first model show that maths anxiety (OR = 0.78, 95% CI [0.64, 0.96], *p* = 0.02) and maths interest (OR = 1.75, 95% CI [1.44, 2.67], *p* < 0.001) significantly and independently predicted STEM career choice. Maths self-efficacy did not independently predict STEM career choice (OR = 1.25, 95% CI [0.96, 1.62], *p* = 0.09). Maths interest remained a significant independent predictor of STEM career choice when both maths achievement and SES were adjusted for, although its effects were attenuated (OR = 1.65, 95% CI [1.33, 2.04], *p* < 0.001). Results from the second model indicate that maths anxiety does not independently predict STEM career choice when the effects of maths achievement are controlled. However, effect sizes in the adjusted models were only slightly attenuated compared to the unadjusted model (ORs ranging between 0.79 and 0.81).

### Sex differences in the joint influence of maths anxiety, maths self-efficacy, and maths interest on STEM career choice

**Fig. 3:**
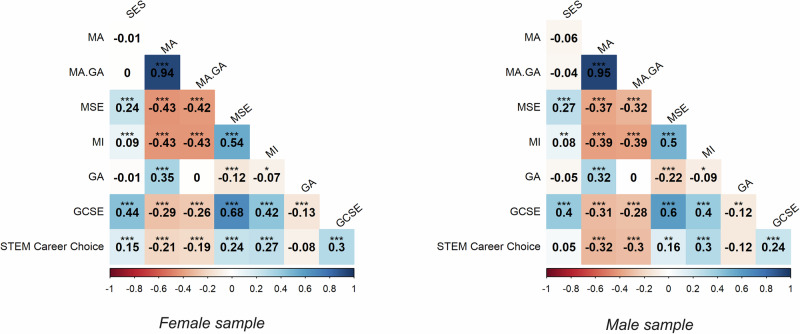
Sex-stratified correlations. Zero-order correlations between all variables in sex-stratified samples. MA maths anxiety, MA.GA maths anxiety (independent of general anxiety), MSE maths self-efficacy, MI maths interest, GA general anxiety, GCSE maths achievement, SES socioeconomic status. *p < 0.05 **p < 0.01, ***p < 0.001.

Figure 3 presents the sex-stratified Pearson’s correlations between all variables. Supplementary Table 8 presents the sample size and 95% confidence intervals for all correlations. The associations between maths interest and STEM career choice were similar in male and female samples (*r* = 0.27 and *r* = 0.30, respectively). The association between maths self-efficacy was greater in the female sample (*r* = 0.24, 95% CI [0.17, 0.31]) than in the male sample (*r* = 0.16, 95% CI [0.06, 0.25]), although overlapping confidence interval suggests this difference is not significant. A small negative correlation was observed between maths anxiety and STEM career choice in the female sample (*r* = −0.21, 95% CI [−0.28, −0.13]). In contrast, the negative correlation was moderate in the male sample (*r* = −0.32, 95% CI [−0.42, −0.21]), although the overlapping confidence intervals again demonstrate that this difference was not significant. Socioeconomic status (SES) was associated with STEM career choice in the female sample (*r* = 0.15, 95% CI [0.10, 0.20]) but not in the male sample (*r* = 0.05, 95% CI [−0.02, 0.11]), although the overlapping confidence intervals show that the effect sizes were not significantly different from each other.

To further investigate sex differences in the joint influence of maths anxiety and maths motivation on STEM career choice, we repeated the three binary logistic regression models in sex-stratified samples. As general anxiety was not significantly associated with STEM career choice in either sample, the unadjusted maths anxiety variable was used for both samples. Variance inflation factors (VIF) for predictor variables in each sample were all less than 1.6, meeting the logistic regression assumption of low or no multicollinearity between predictors (see Supplementary Tables 9 and 10 for full VIF for each predictor variable).

**Fig. 4:**
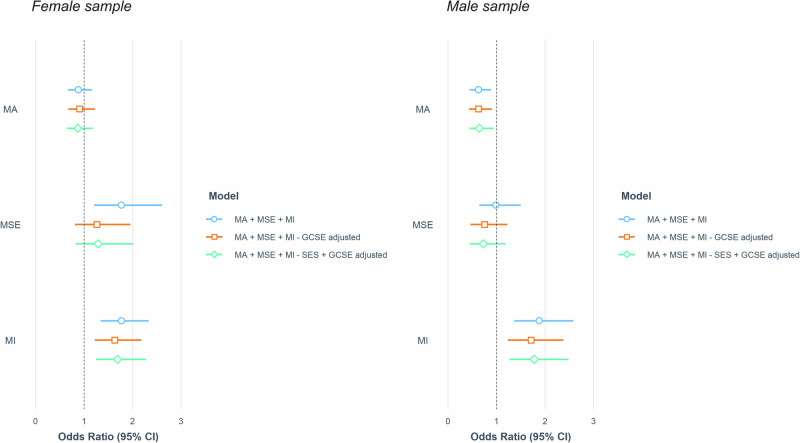
Sex-stratified associations between maths anxiety, maths self-efficacy and maths interest and STEM career choice. Comparison of the joint effects of maths anxiety, maths self-efficacy, and maths interest on STEM career choice in sex-stratified logistic regression models. MA maths anxiety, MSE maths self-efficacy, MI maths interest, GCSE GCSE maths achievement, SES socioeconomic status.

Supplementary Table 11 summarises the effects observed for the logistic regression models in the sex-stratified samples. Figure 4 compares the effects of maths anxiety, maths self-efficacy and maths interest on STEM career choice in male and female samples across all three models. The Nagelkerke pseudo-R-squared for models in the female sample ranged between 0.16 and 0.23. In the female sample, maths interest significantly predicted STEM career choice across all models (ORs ranging from 1.63 to 1.77) and maths self-efficacy predicted STEM career choice in the unadjusted model only (OR = 1.77). Maths anxiety did not independently predict STEM career choice in any model performed in the female sample. In the male sample, the Nagelkerke pseudo-R-squared ranged between 0.18 and 0.19. Both maths interest (ORs ranging from 1.71 to 1.88) and maths anxiety (ORs ranging from 0.62 to 0.64) predicted STEM career choice in every model in the male sample. Maths self-efficacy did not predict STEM career choice in any model performed in the male sample.

Maths anxiety was an independent predictor of STEM career choice in the unadjusted male model (OR = 0.62, 95% CI [0.42, 0.89]) but not in the unadjusted female model (OR = 0.88, 95% CI [0.67, 1.16]). A Wald Chi-square test showed that maths anxiety was not a significantly better predictor of STEM career choice in the male sample than in the female sample (X^2^ = 2.26, *p* = 0.13). However, the effect of maths self-efficacy in the unadjusted female model (OR = 1.77, 95% CI [1.20, 2.60]) was larger than the effect observed in the unadjusted male model (OR = 0.98, 95% CI [0.65, 1.50]), and this difference was found to be significant (X^2^ = 4.07, *p* = 0.043).

## Discussion

Previous research has highlighted the influence of maths anxiety, maths self-efficacy and maths interest on STEM participation^9,11–13,35^ but has not considered the joint effects of these factors on individuals’ STEM career choices. The results of the present study suggest that maths anxiety and maths interest in adolescence independently predict STEM career choices in emerging adulthood. We also showed that while the independent effects of maths interest remained significant when both SES and maths achievement were accounted for, the already weak effect of maths anxiety no longer predicted STEM career choice after accounting for maths achievement. This finding challenges the prevalent narrative that maths anxiety invariably leads to STEM avoidance^11,12,40^, and underscores the complexity of the pathway from maths anxiety to STEM career choice. However, it is consistent with existing work considering the joint effects of maths anxiety and maths motivational attitudes on general STEM participation^31^.

Maths self-efficacy at age 16 did not independently predict STEM career choice in our study. This finding contrasts with the expectations of Social Cognitive Career Theory (SCCT), which proposes that self-efficacy in a given domain is a key driver for later career-related actions^14^. However, we observed a significant and substantial correlation between maths self-efficacy and maths interest (*r* = 0.52). A longitudinal study of students in primary education has demonstrated that prior maths self-efficacy predicts later maths interest^3^, suggesting that, consistent with SCCT, self-efficacy may impact later STEM career outcomes indirectly through its influence on developing interest. The same study also showed that prior maths anxiety also predicted later maths interest^3^, indicating that maths anxiety may also further impact STEM career choice through its impact on maths interest earlier in development. Although our measures of maths self-efficacy, maths interest, and maths anxiety were taken at a single time point, these relationships highlight the need for longitudinal research over adolescence and early adulthood to fully understand how these constructs interact over time to influence STEM career choices in emerging adulthood.

Our results provide some support for the prediction that maths anxiety might be more decisive for STEM career choice in males than in females^30^, as this study found that maths anxiety was a significant predictor of STEM career choice in the male sample only. This is consistent with a recent study that found that maths anxiety predicted male STEM participation to a greater extent than it did female STEM participation^12^. Male students have been shown to identify with maths more than females and feel more pressure to perform well^41^, so high maths anxiety might threaten their identity more, potentially leading to reduced STEM participation. However, we also observed that the difference in the effects of maths anxiety by sex was non-significant, suggesting that the evidence for this sex-specific impact is weak.

We observed that maths interest was a robust predictor of STEM career choice for both males and females, but this investigation also found that maths self-efficacy was a significant predictor of STEM career choice in the female, but not male sample. This is consistent with a previous study, which also found that maths self-efficacy predicted STEM participation in females but not males^13^ and suggests that self-perceptions of competency are more important for female STEM career participation than male. However, we also found that this effect was attenuated when accounting for maths achievement, showing that the independent association between maths self-efficacy and female STEM participation can be explained by maths achievement levels.

Although only slight sex differences were observed in the joint effects of maths anxiety, maths interest and maths self-efficacy on STEM career choice, analyses of mean-level differences demonstrate that consistent with previous research^12,35,36^, male participants are more than twice as likely to choose a STEM career than female participants. Sex accounts for only 0.1% of the variance in maths achievement, suggesting that sex differences in maths achievement are unlikely to account for much of this difference. Females had significantly higher maths anxiety, with sex explaining 6% of the variance, in line with prior work^11,16,24^. Although higher female maths anxiety is thought to reduce STEM participation^11,42,43^, the weak effects of this construct on STEM career choice demonstrated here, particularly in the female-only sample, suggest that this may not be the case. Conversely, consistent with previous studies^13,33,34^, maths interest was found to be significantly higher in males, with sex explaining 1% of the variance. Maths interest was also found to be a robust predictor of STEM career choice in both male and female samples, making it a stronger potential candidate than maths anxiety for at least partially explaining the large sex disparity in STEM career choice.

There are some limitations to this study. Robustness checks (see Supplementary Table 12) demonstrated that data missing across all variables were from individuals with a lower mean SES level than those who contributed data. In particular, the maths achievement and STEM career choice variables were shown to have a moderate effect of SES on missing data. This issue is likely to be further affected by the career data availability being limited to degree and apprenticeship decisions, as previous work demonstrates that these intakes tend to favour individuals from higher SES backgrounds in the UK^44^. While we observed a small effect of SES on STEM career choice, our investigation also highlighted that SES had little attenuating effect on the influence of maths anxiety, maths self-efficacy and maths interest on STEM career choice beyond that of maths achievement. It is possible that this may not be the case for individuals who come from particularly low SES backgrounds. Replicating the results presented in this study in populations with a full range of SES, perhaps by using data for career choices beyond degrees and apprenticeships, would allow for greater generalisability.

A further limitation of this study is that the measures of maths self-efficacy and maths interest were taken two years earlier than the maths anxiety measure. However, longitudinal research has found moderate to high levels of stability in maths interest, maths self-efficacy^2,45^ and maths anxiety^46^, suggesting that this two-year time lapse may not significantly impact the findings. Nonetheless, as mentioned earlier, it is crucial to replicate these findings with a more comprehensive understanding of how maths anxiety, maths self-efficacy and maths interest develop over adolescence and into early adulthood.

Finally, while the associations between maths anxiety, maths self-efficacy, maths interest and STEM career choice are evident, this study design does not allow for causal inference. The observed relationships between these variables could be influenced by a third variable that this investigation has not accounted for. It has been suggested that emotional and motivational factors specific to STEM more broadly, rather than maths only (e.g., STEM anxiety and STEM interest and self-efficacy), may also influence the likelihood of STEM career choices^21,24^. Additionally, the STEM anxiety framework^24^ highlights how wider social, emotional and motivational factors, such as parental influence, school or teacher influence, financial or career motivation levels, and self-regulation ability, are likely to have important roles to play. Future research should consider whether these proposed factors change the associations between maths anxiety, maths self-efficacy, maths interest and STEM career choice.

In conclusion, our study adds to our understanding of how maths-related motivational and emotional factors in adolescence contribute to STEM career choice in emerging adulthood. It highlights the potential of enhancing maths interest in adolescence over merely reducing maths anxiety and increasing self-efficacy, especially considering the effect of maths interest for both males and females. Our findings suggest developing targeted interventions that foster STEM participation, aligning with the UK’s economic need for increased STEM expertise. The complex interplay of maths anxiety and maths motivational attitudes and their development over time warrants further longitudinal research to accurately inform targeted educational strategies and policies.

## Methods

### Participants

Participants were from the Twins Early Development Study (TEDS), a longitudinal study of twins born between 1994 and 1996 in England and Wales. TEDS participants were representative of the wider population of England and Wales in terms of ethnicity and socioeconomic status at conception, and although there has been some attrition over time (a 76.5% response rate by early adulthood), the sample remains representative of the birth cohort. More detailed information on the TEDS sample is available elsewhere^47^. Informed consent was obtained from participants at each wave of data collection, and the study received ethical approval from the King’s College London Ethics Committee (PNM/09/10-104). This investigation used data collected in a subsample of TEDS twins over four waves: first contact (when twins were ~1.5 years old), age ~16 (mean = 16.31, SD = 0.29), age ~18–21 (mean = 20.30, SD = 0.48), and age ~21–24 (mean = 22.28, SD = 0.91). One twin from each pair was randomly selected for analyses to control for non-independence of observations.

At first contact, the parents of twin participants contributed data on their families’ socioeconomic status (SES; *N* = 7345, 53% female). SES was taken at first contact as this wave offered minimal missing data, and SES has been shown to be developmentally stable, with age-to-age correlations ~0.70^48^. At age 16, participants contributed data on maths achievement (*N* = 6409, 52% female) and maths self-efficacy and interest (*N* = 2558, 58% female). At age 18–21, data on general anxiety and maths anxiety were collected from 2 out of 4 TEDS cohorts (*N* = 1489, 64% female). At age 21–24, participants contributed data on their choice of degree and/or apprenticeship (*N* = 2254, 64% female). 94.8% (2137) of individuals reported a degree choice only, and 4.7% (105) reported an apprenticeship choice only. 0.5% (12) reported both a degree and an apprenticeship choice. Sample sizes varied across variables due to participation levels and data collection differences across waves. Individuals with major medical, neurodevelopmental or genetic disorders were excluded from the study sample.

### Missing data analysis

Robustness checks indicated MNAR patterns in our data, as individuals with missing data in our longitudinal study had lower socioeconomic status (SES) than those with complete data, consistent with the literature on drop-out predictors^49^. Independent t-tests across all constructs confirmed these SES differences, as reported in Supplementary Table 12, alongside construct-level missingness percentages. Data contributors had significantly higher SES, with its absence accounting for 7–14% of the variance in anxiety, self-efficacy and interest measures and a substantial 43–44% in maths achievement and STEM career choice. Missing data led to the exclusion of affected individuals from the analysis, with the impact of SES on missingness duly considered in our interpretations.

### Key variables

#### Mathematics self-efficacy and interest

Two scales adapted from the OECD Programme for International Student Assessment measured mathematics interest and self-efficacy. The maths interest scale consisted of three items: ‘I look forward to my mathematics lessons’, ‘I do mathematics because I enjoy it’ and ‘I am interested in the things I learn in mathematics’. The items had to be rated on a four-point scale from 1 (strongly disagree) to 4 (strongly agree). The scale showed good internal validity (α = 0.93). The mathematics self-efficacy scale asked, ‘How confident do you feel having to do the following tasks’ and consisted of eight items rated on a four-point scale from 0 (not at all confident) to 3 (very confident). Items included ‘solving an equation like 2(x + 3) = (x + 3)(x − 3)’ and ‘calculating how much cheaper a TV would be after a 30% discount’. This scale also showed good internal validity (α = 0.90).

#### Mathematics anxiety

Mathematics anxiety was assessed using an adapted version of the Abbreviated Math Anxiety Scale (AMAS)^50^. The scale asks participants to rate their anxiety level when facing a range of maths-related situations. Participants rated nine items on a five-point scale, from ‘not nervous at all’ to ‘very nervous’. Items included ‘listening to someone explain a maths formula’, ‘reading a maths book’ and ‘taking an examination in a maths course’. The AMAS has good internal validity (α = 0.94) and test-retest reliability (*r* = 0.85)^50^.

#### General anxiety

The Generalised Anxiety Disorder Scale (GAD-7) was used to collect data on general anxiety^51^. The scale asks participants to rate seven items on a four-point scale from 1 (not at all) to 4 (nearly every day), ‘how often in the past month have you been bothered by the following problems?’. Items include ‘feeling nervous, anxious or on edge’, ‘being so restless that it is hard to sit still’ and ‘feeling afraid as if something awful might happen’. The GAD-7 has good internal validity (α = 0.89) and moderate test-retest reliability (*r* = 0.64).

#### Maths anxiety (independent of general anxiety)

The maths anxiety measure (described above) was residualised for general anxiety (also described above) in the study sample to create a maths anxiety variable that was independent of the effects of general anxiety.

#### STEM career choice

At age 21, participants self-reported the degree subject they were either studying or had completed or the apprenticeship that they were either actively working in or had completed. 46.5% (2254) of participants at age 21 reported making a degree and/or apprenticeship choice. Degree and apprenticeship career routes were combined in this study, as only 117 (5.2%) of the cohort had chosen an apprenticeship route. Participants’ fields of work and study were classified as STEM or non-STEM using the UK’s Higher Education Statistics Agency (HESA) subject coding system—the Higher Education Classification of Subjects (HECoS)^52^. STEM career classifications included (but were not limited to) Mathematical Sciences, Computer Sciences and Technology, Medicine and allied professions, and Veterinary Sciences and Agriculture. Non-STEM career classification categories included (but were not limited to) Law, Creative Arts and Design, and Business Administration. STEM career choice was coded as a dichotomous variable where 1 = STEM career choice and 0 = Non-STEM career choice. The 0.5% of participants who had reported both a degree and apprenticeship choice were considered to have made a STEM career choice if one or both choices met the STEM career classification criteria.

### Covariates

#### Maths achievement

Students take General Certificate of Secondary Education (GCSE) exams across all subjects at the end of compulsory education in the United Kingdom, at age 15–16. The mathematics GCSE exam is a mandatory part of the National Curriculum. Grades were obtained from questionnaires completed by parents or the twins themselves or via phone or email contact. Grades were recorded on a scale from 11 (corresponding to A*, the highest possible score) to 4 (corresponding to G, the lowest possible score). For a subsample of twins, self- and parent-reported GCSE grades were verified using data from the National Pupil Database (NPD)^53^, resulting in a correlation of 0.99 for mathematics^54^.

#### Socioeconomic status (SES)

Family SES was assessed at first contact. Mothers and fathers reported their educational level, occupational status and mothers’ age at the birth of their first child. The composite of SES was computed as the mean of the standardised scores of these measures. Socioeconomic status (SES) was taken at first contact as this wave offers minimal missing data, and SES has been shown to be developmentally stable, with age-to-age correlations of ~0.70^48^.

### Statistical analyses

**Fig. 5:**
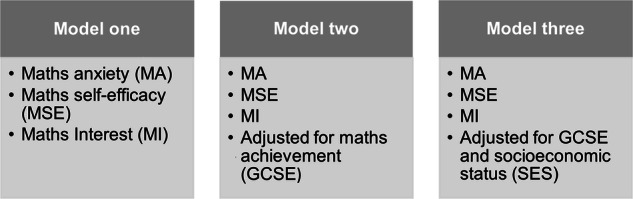
Logistic regression models. The three binary logistic regression models performed in the current study.

All analyses were conducted in R version 4.2.3. First, descriptive analyses were calculated for all variables. Univariate ANOVAs were used to investigate the mean-level sex differences for all variables. Then, zero-order correlations were carried out in the whole study sample to determine the relationships between all variables. Next, three binary logistic regression models were performed to determine the joint influence of mathematics anxiety (both corrected and uncorrected for general anxiety), maths self-efficacy, maths interest on STEM career choice and to investigate the effects of adjusting for previous maths achievement and socioeconomic status, as illustrated by Fig. 5. All continuous variables were residualised for age and sex, and these were used for all downstream analyses.

The zero-order correlations between all variables and binary logistic regressions outlined in Fig. 5 were then repeated in sex-stratified samples to assess whether the joint influence of maths anxiety, maths self-efficacy, and maths interest on STEM career choice differs between males and females. In the sex-stratified analyses, all continuous variables were residualised for age only before testing.

## Supplementary information

Supplementary Material

## Data Availability

Data for this study came from the Twins Early Development Study (TEDS). Data is available to researchers upon request. For more information on data availability, please see the TEDS data access policy: https://www.teds.ac.uk/researchers/teds-data-access-policy.

## Code availability

Code used for this study will be made available in the Open Science Framework registry (OSF; https://osf.io/y36bg).

## References

[1] Regan, E. & DeWitt, J. Attitudes, interest and factors influencing STEM enrolment behaviour: an overview of relevant literature in *Understanding Student Participation and Choice in Science and Technology Education* (eds. Henriksen, E. K. et al.) 63–88 (Springer, 2015).

[2] Grigg, S., Perera, H. N., McIlveen, P. & Svetleff, Z. Relations among math self efficacy, interest, intentions, and achievement: a social cognitive perspective. *Contemp. Educ. Psychol.* **53**, 73–86 (2018).

[3] Du, C., Qin, K., Wang, Y. & Xin, T. Mathematics interest, anxiety, self-efficacy and achievement: examining reciprocal relations. *Learn. Individ. Differ.* **91**, 102060 (2021).

[4] Institution of Engineering and Technology. *IET Skills and Demand in Industry 2021 Survey*. https://www.theiet.org/impact-society/factfiles/innovation-and-skills-factfiles/uk-skills-surveys/skills-survey-2021/ (2021).

[5] House of Lords Science and Technology Committee. *S**cience and Technology Committee*—*Inquiry into People and Skills in UK STEM. UK Parliament*. https://committees.parliament.uk/publications/33254/documents/179987/default/ (2022).

[6] British Science Association. *I**nquiry into Equity in the STEM Workforce: Final Report*. https://www.britishscienceassociation.org/news/report-on-equity-in-stem-workforce-published (2021).

[7] Halpern, D. F. Science, sex, and good sense: why women are underrepresented in some areas of science and math in *Why Aren’t More Women in Science? Top Researchers Debate the Evidence*, 121–130 (American Psychological Association, 2007).

[8] Wai, J., Putallaz, M. & Makel, M. Studying intellectual outliers: are there sex differences, and are the smart getting smarter? *Curr. Dir. Psychol. Sci*. **21**, 382–390 (2012).

[9] Guo, J., Parker, P. D., Marsh, H. W. & Morin, A. J. S. Achievement, motivation, and educational choices: a longitudinal study of expectancy and value using a multiplicative perspective. *Dev. Psychol.* **51**, 1163–1176 (2015). 26053150 10.1037/a0039440

[10] Maltese, A. V. & Tai, R. H. Pipeline persistence: examining the association of educational experiences with earned degrees in STEM among U.S students. *Sci. Educ.* **95**, 877–907 (2011).

[11] Ahmed, W. Developmental trajectories of math anxiety during adolescence: associations with STEM career choice. *J. Adolesc.* **67**, 158–166 (2018). 29975882 10.1016/j.adolescence.2018.06.010

[12] Daker, R. J., Gattas, S. U., Sokolowski, H. M., Green, A. E. & Lyons, I. M. First-year students’ math anxiety predicts STEM avoidance and underperformance throughout university, independently of math ability. *NPJ Sci. Learn.* **6**, 1–13 (2021). 34127672 10.1038/s41539-021-00095-7 PMC8203776

[13] Watt, H. et al. Mathematics—a critical filter for STEM-related career choices? A longitudinal examination among Australian and U.S. adolescents. *Sex Roles* **77**, 254–271 (2017).

[14] Lent, R. W., Brown, S. D. & Hackett, G. Toward a unifying social cognitive theory of career and academic interest, choice, and performance. *J. Vocat. Behav.* **45**, 79–122 (1994).

[15] Brown, D. *Career Choice and Development* (Wiley, 2002).

[16] Malanchini, M. et al. Genetic factors underlie the association between anxiety, attitudes and performance in mathematics. *Transl. Psychiatry* **10**, 1–11 (2020). 32066693 10.1038/s41398-020-0711-3 PMC7026074

[17] Lee, W., Lee, M.-J. & Bong, M. Testing interest and self-efficacy as predictors of academic self-regulation and achievement. *Contemp. Educ. Psychol.* **39**, 86–99 (2014).

[18] Sakellariou, C. The reciprocal relationship between mathematics self-efficacy and mathematics performance in US high school students: instrumental variables estimates and gender differences. *Front. Psychol*. **13**, 941253 (2022). 10.3389/fpsyg.2022.941253 PMC9535693 36211864

[19] Yang, Y., Maeda, Y. & Gentry, M. The relationship between mathematics self-efficacy and mathematics achievement: multilevel analysis with NAEP 2019. *Large-Scale Assess. Educ.* **12**, 16 (2024).

[20] Wiederkehr, V., Darnon, C., Chazal, S., Guimond, S. & Martinot, D. From social class to self-efficacy: internalization of low social status pupils’ school performance. *Soc. Psychol. Educ. Int. J.* **18**, 769–784 (2015).

[21] Jiang, S., Simpkins, S. D. & Eccles, J. S. Individuals’ math and science motivation and their subsequent STEM choices and achievement in high school and college: a longitudinal study of gender and college generation status differences. *Dev. Psychol.* **56**, 2137–2151 (2020). 32915052 10.1037/dev0001110

[22] Chiu, L.-H. & Henry, L. Development and validation of the mathematics anxiety scale for children. *Meas. Eval. Couns. Dev.* **23**, 121–127 (1990).

[23] Bai, H., Wang, L., Pan, W. & Frey, M. Measuring mathematics anxiety: psychometric analysis of a bidimensional affective scale. *J. Instr. Psychol.* **36**, 185–193 (2009).

[24] Grimes, Z. & Gardner, G. Conceptions of disciplinary anxiety across science, technology, engineering, and mathematics (STEM) contexts: a critical and theoretical synthesis. *J. Res. Sci. Math. Technol. Educ.* **6**, 21–46 (2023).

[25] McLean, L., Janssen, J., Espinoza, P., Lindstrom Johnson, S. & Jimenez, M. Associations between teacher and student mathematics, science, and literacy anxiety in fourth grade. *J. Educ. Psychol.* **115**, 539–551 (2023).

[26] Živković, M. et al. Math self-efficacy or anxiety? The role of emotional and motivational contribution in math performance. *Soc. Psychol. Educ*. 10.1007/s11218-023-09760-8 (2023).

[27] Wang, Z., Shakeshaft, N., Schofield, K. & Malanchini, M. Anxiety is not enough to drive me away: a latent profile analysis on math anxiety and math motivation. *PLoS ONE* **13**, e0192072 (2018). 29444137 10.1371/journal.pone.0192072 PMC5812593

[28] Wang, Z. et al. Is math anxiety always bad for math learning? The role of math motivation. *Psychol. Sci.* **26**, 1863–1876 (2015). 26518438 10.1177/0956797615602471 PMC4679544

[29] Levy, H. E., Fares, L. & Rubinsten, O. Math anxiety affects females’ vocational interests. *J. Exp. Child Psychol.* **210**, 105214–105214 (2021). 34198037 10.1016/j.jecp.2021.105214

[30] Wang, Z., Oh, W., Malanchini, M. & Borriello, G. A. The developmental trajectories of mathematics anxiety: cognitive, personality, and environmental correlates. *Contemp. Educ. Psychol.* **61**, 101876 (2020).

[31] Meece, J. L., Eccles, J. S. & Wigfield, A. Predictors of math anxiety and its influence on young adolescents’ course enrollment intentions and performance in mathematics. *J. Educ. Psychol*. **82**, 60–70 (1990).

[32] Wang, M.-T. & Degol, J. Motivational pathways to STEM career choices: using expectancy–value perspective to understand individual and gender differences in STEM fields. *Dev. Rev.* **33**, 304–340 (2013). 10.1016/j.dr.2013.08.001 PMC3843492 24298199

[33] Reilly, D., Neumann, D. L. & Andrews, G. Investigating gender differences in mathematics and science: results from the 2011 Trends in Mathematics and Science Survey. *Res. Sci. Educ.* **49**, 25–50 (2019).

[34] Rodríguez, S., Regueiro, B., Piñeiro, I., Estévez, I. & Valle, A. Gender differences in mathematics motivation: differential effects on performance in primary education. *Front. Psychol*. **10**, 3050 (2020). 10.3389/fpsyg.2019.03050 PMC7000542 32063870

[35] Wang, M.-T., Degol, J. & Ye, F. Math achievement is important, but task values are critical, too: examining the intellectual and motivational factors leading to gender disparities in STEM careers. *Front. Psychol*. **6**, 36 (2015). 10.3389/fpsyg.2015.00036 PMC4330678 25741292

[36] Wang, M.-T. & Degol, J. L. Gender gap in science, technology, engineering, and mathematics (STEM): current knowledge, implications for practice, policy, and future directions. *Educ. Psychol. Rev.* **29**, 119–140 (2017). 28458499 10.1007/s10648-015-9355-x PMC5404748

[37] Wang, Z., Rimfeld, K., Shakeshaft, N., Schofield, K. & Malanchini, M. The longitudinal role of mathematics anxiety in mathematics development: issues of gender differences and domain-specificity. *J. Adolesc.* **80**, 220–232 (2020). 32199102 10.1016/j.adolescence.2020.03.003

[38] Hyde, J. S. Gender similarities and differences. *Annu. Rev. Psychol.* **65**, 373–398 (2014). 23808917 10.1146/annurev-psych-010213-115057

[39] Priess-Groben, H. A. & Hyde, J. S. Implicit theories, expectancies, and values predict mathematics motivation and behavior across high school and college. *J. Youth Adolesc.* **46**, 1318–1332 (2017). 27681409 10.1007/s10964-016-0579-y

[40] Hembree, R. The nature, effects, and relief of mathematics anxiety. *J. Res. Math. Educ.* **21**, 33–46 (1990).

[41] Cvencek, D., Meltzoff, A. N. & Greenwald, A. G. Math–gender stereotypes in elementary school children. *Child Dev.* **82**, 766–779 (2011). 21410915 10.1111/j.1467-8624.2010.01529.x

[42] Ashcraft, M. H. & Krause, J. A. Working memory, math performance, and math anxiety. *Psychon. Bull. Rev.* **14**, 243–248 (2007). 17694908 10.3758/bf03194059

[43] Devine, A., Fawcett, K., Szűcs, D. & Dowker, A. Gender differences in mathematics anxiety and the relation to mathematics performance while controlling for test anxiety. *Behav. Brain Funct.* **8**, 33 (2012). 22769743 10.1186/1744-9081-8-33 PMC3414752

[44] Cavaglia, C., Ventura, G. & McNally, S. *The Recent Evolution of Apprenticeships*. https://www.suttontrust.com/our-research/the-recent-evolution-of-apprenticeships/ (2022).

[45] Luo, Y. L. L., Kovas, Y., Haworth, C. M. A. & Plomin, R. The etiology of mathematical self-evaluation and mathematics achievement: understanding the relationship using a cross-lagged twin study from age 9 to 12. *Learn. Individ. Differ.* **21**, 710–718 (2011). 22102781 10.1016/j.lindif.2011.09.001 PMC3217262

[46] Ma, X. & Xu, J. The causal ordering of mathematics anxiety and mathematics achievement: a longitudinal panel analysis. *J. Adolesc.* **27**, 165–179 (2004). 15023516 10.1016/j.adolescence.2003.11.003

[47] Rimfeld, K. et al. Twins early development study: a genetically sensitive investigation into behavioral and cognitive development from infancy to emerging adulthood. *Twin Res. Hum. Genet.* **22**, 508–513 (2019). 31544730 10.1017/thg.2019.56 PMC7056571

[48] Hanscombe, K. B. et al. Socioeconomic status (SES) and children’s intelligence (IQ): in a UK-representative sample SES moderates the environmental, not genetic, effect on IQ. *PLoS ONE* **7**, e30320 (2012). 22312423 10.1371/journal.pone.0030320 PMC3270016

[49] Schmidt, S. C. E. & Woll, A. Longitudinal drop-out and weighting against its bias. *BMC Med. Res. Methodol.* **17**, 164 (2017). 29221434 10.1186/s12874-017-0446-x PMC5723086

[50] Hopko, D. R., Mahadevan, R., Bare, R. L. & Hunt, M. K. The Abbreviated Math Anxiety Scale (AMAS): construction, validity, and reliability. *Assessment* **10**, 178–182 (2003). 12801189 10.1177/1073191103010002008

[51] Löwe, B. et al. Validation and standardization of the Generalized Anxiety Disorder Screener (GAD-7) in the general population. *Med. Care* **46**, 266–274 (2008). 18388841 10.1097/MLR.0b013e318160d093

[52] Higher Education Statistics Agency. *The Higher Education Classification of Subjects (HECoS)*. https://www.hesa.ac.uk/support/documentation/hecos (2020).

[53] Jay, M. A., Grath-Lone, L. M. & Gilbert, R. Data resource: the National Pupil Database (NPD). *Int. J. Popul. Data Sci*. **4**, 1101 (2019). 10.23889/ijpds.v4i1.1101 PMC7482519 32935030

[54] Krapohl, E. et al. The high heritability of educational achievement reflects many genetically influenced traits, not just intelligence. *Proc. Natl Acad. Sci. USA* **111**, 15273–15278 (2014). 25288728 10.1073/pnas.1408777111 PMC4210287

