# Supplementary material for: Mathematics interest, self-efficacy, and anxiety predict STEM career choice in emerging adulthood
Source: NPJ Sci Learn. 2024 Nov 13;9:66. doi: 10.1038/s41539-024-00275-1 (PMC11561120; doi:10.1038/s41539-024-00275-1)
Supplement: Supplementary file 1 — Supplementary Material [file 41539_2024_275_MOESM1_ESM.docx]

# Supplementary materials

**Mathematics interest, self-efficacy, and anxiety predict STEM career choice in emerging adulthood**

**Rebecca Ferdinand^1*^, Margherita Malanchini^2,3^, and Kaili Rimfeld^1,3^**

^1^ Department of Psychology, Royal Holloway, University of London, UK

^2^ School of Biological and Behavioural Sciences, Queen Mary University of London, UK

^3^ Social, Genetic and Developmental Psychiatry Centre, Institute of Psychiatry, Psychology and Neuroscience, King's College London, UK

*Corresponding author at rebecca.ferdinand@rhul.ac.uk

Table of Contents

[Supplementary materials 1](#_Toc175748370)

[Supplementary Table 1: Descriptive statistics for all continuous variables. 2](#_Toc175748371)

[Supplementary Table 2: Frequency count and proportions of STEM career choice variable. 2](#_Toc175748372)

[Supplementary Table 3: Univariate analyses of variance (ANOVA) examining sex differences in all continuous variables 3](#_Toc175748373)

[Supplementary Table 4: Contingency table for sex differences in STEM career choice 3](#_Toc175748374)

[Supplementary Table 5: Zero-order correlations between all variables in the whole sample *(N; 95% CIs)* 4](#_Toc175748375)

[Supplementary Table 6: Variance inflation factors (VIFs) for predictor variables in each of the four binary logistic regression models used in the whole sample. 5](#_Toc175748376)

[Supplementary Table 7: Binary logistic regression models assessing the joint influence of maths anxiety and maths motivation on STEM career choice. 6](#_Toc175748377)

[Supplementary Table 8: Zero-order correlations between all variables in sex-stratified samples *(N; 95% CIs)* 7](#_Toc175748378)

[Supplementary Table 9: Variance inflation factors (VIFs) for predictor variables in each of the four binary logistic regression models used in the female sample 9](#_Toc175748379)

[Supplementary Table 10: Variance inflation factors (VIFs) for predictor variables in each of the four binary logistic regression models used in the male sample. 9](#_Toc175748380)

[Supplementary Table 11: Binary logistic regression models assessing the joint influence of maths anxiety and maths motivation on STEM career choice in sex-stratified samples. 10](#_Toc175748381)

[Supplementary Table 12: T-test results for the robustness checks on the socioeconomic status in participating compared to non- participating individuals. 11](#_Toc175748382)

|  | Supplementary Table 1: Descriptive statistics for all continuous variables. | | | | | | | |
| --- | --- | --- | --- | --- | --- | --- | --- | --- |
|  | | Maths anxiety | General anxiety | Maths achievement (GCSE) | Maths interest | Maths self-efficacy | | SES* |
| N | | 1489 | 1489 | 6409 | 2559 | 2559 | 7435 | |
| Mean | | 2.26 | 1.96 | 8.9 | 2.54 | 17.72 | 0.16 | |
| St Dev | | 0.98 | 0.74 | 1.44 | 0.94 | 5.36 | 0.99 | |
| SE | | 0.03 | 0.02 | 0.02 | 0.02 | 0.11 | 0.01 | |
| Skew | | 0.74 | 0.89 | -0.57 | -0.09 | -0.84 | -0.07 | |
| Kurtosis | | -0.19 | 0.12 | 0.37 | -0.99 | 0.12 | -0.77 | |
| Minimum | | 1 | 1 | 4 | 1 | 0 | -2.46 | |
| Maximum | | 5 | 4 | 11 | 4 | 24 | 2.65 | |
|  | Note: one twin out of each pair was randomly selected to control for non-independence of observations.  SE – standard error; St Dev- standard deviation  *Standardised variable – see methods section for details | | | | | | | |

| Supplementary Table 2: Frequency count and proportions of STEM career choice variable. | | | |
| --- | --- | --- | --- |
|  | Total N | Frequency Count | |
| STEM Career Choice | 2254 | STEM  662 | Non-STEM  1592 |
|  |  | Proportion (%) | |
|  |  | STEM  29.4% | Non-STEM  70.6% |
| Note: one twin out of each pair was selected to control for non-independence of observation. | | | |

| Supplementary Table 3: Univariate analyses of variance (ANOVA) examining sex differences in all continuous variables. | | | | |
| --- | --- | --- | --- | --- |
|  | Female M (SD), N | Male M (SD), N | F | Partial η2 |
| Maths anxiety | 2.43 (1.02), N = 948 | 1.96 (0.82), N = 541 | 86.68*** | 0.06 |
| General anxiety | 2.06 (0.77), N = 948 | 1.77 (0.62), N = 541 | 57.74*** | 0.04 |
| Maths achievement (GCSE) | 8.85 (1.44), N = 3359 | 8.96 (1.44), N = 3050 | 9.18** | 0.001 |
| Maths self-efficacy | 16.65 (5.49), N = 1485 | 19.2 (4.81), N = 1074 | 149.10*** | 0.05 |
| Maths interest | 2.45 (0.95), N = 1485 | 2.68 (0.89) N = 1074 | 39.04*** | 0.01 |
| SES | 0.13 (0.99), N = 3923 | 0.18 (0.99), N = 3512 | 5.05* | 0.0007 |
| Note: one twin out of each pair was selected to control for non-independence of observation  * = p < .05 ** = p< .01, *** = p<.001  M - mean  SD – standard deviation | | | | |

| Supplementary Table 4: Contingency table for sex differences in STEM career choice. | | |
| --- | --- | --- |
|  | STEM career choice – % (Frequency) | Non-STEM career choice % (Frequency) |
| Female  Total N = 1440  (63.9%) | 21.9% (316) | 78.1% (1124) |
| Male  Total N = 814  (31.9%) | 42.5% (346) | 57.5% (468) |
| Note: one twin out each pair was randomly selected to control for non-independence of observation. | | |

|  | Supplementary Table 5: Zero-order correlations between all variables in the whole sample *(N; 95% CIs)* | | | | | | |
| --- | --- | --- | --- | --- | --- | --- | --- |
|  | SES | MA | MA (independent of GA) | GA | MSE | MI | GCSE |
| SES | ----------- |  |  |  |  |  |  |
| MA | -0.02  (1416; -0.07, 0.03) | ------------ |  |  |  |  |  |
| MA (independent of GA) | -0.01  (1416; -0.07, 0.04) | 0.94***  (1487; 0.93, 0.95) | --------------- |  |  |  |  |
| GA | -0.02  (1416; -0.08, 0.03) | 0.34***  (1487; 0.30, 0.39) | 0.00  (1487; -0.05, 0.05) | ---------- |  |  |  |
| MSE | 0.25***  (2421; 0.21, 0.29) | -0.41***  (1473; -0.52, -0.44) | -0.39***  (1473; -0.51, -0.43) | -0.15***  (1473;  -0.18, -0.07) | --------- |  |  |
| MI | 0.09***  (2422; 0.05, 013) | -0.41***  (1474; -0.46, -0.37) | -0.41***  (1474; -0.45, -0.37) | -0.08**  (1474; -0.13, -0.03) | 0.52***  (2556; 0.49, 0.55) | ------- |  |
| GCSE | 0.42***  (6077; 0.40, 0.44) | -0.29***  (1364;  -0.34, -0.24) | -0.26***  (1364; -0.31, -0.21) | -0.13***  (1364;  -0.18, -0.07) | 0.65***  (2237; 0.62, 0.67) | 0.41***  (2237;0.36, 0.44) | ------------- |
| STEM career choice | 0.12***  (2171; 0.08, 0.17) | -0.23***  (857;  -0.29, -0.17) | -0.21***  (857; -0.27, -0.15) | -0.09*  (857;  -0.15, -0.02) | 0.21***  (1122; 0.15, 0.26) | 0.27***  (1122; 0.21, 0.32) | 0.28***  (1937; 0.24, 0.32) |
|  | Notes: one twin out of each pair was selected to control for non-independence of observation  MA – maths anxiety, MSE – maths self-efficacy, MI – maths interest, GA – general anxiety, GCSE – maths achievement (GCSE), SES – socioeconomic status.  * = p < .05 ** = p< .01, *** = p<.001. | | | | | | |

| Supplementary Table 6: Variance inflation factors (VIFs) for predictor variables in each of the four binary logistic regression models used in the whole sample. | | | |
| --- | --- | --- | --- |
| Predictor variables | Model one | Model two | Model three |
| Maths anxiety | 1.32 | 1.36 | 1.37 |
| Maths self-efficacy | 1.34 | 1.59 | 1.59 |
| Maths interest | 1.30 | 1.33 | 1.33 |
| Maths achievement (GCSE) | - | 1.34 | 1.49 |
| Socioeconomic status (SES) | - | - | 1.15 |

| Supplementary Table 7: Binary logistic regression models assessing the joint influence of maths anxiety and maths motivation on STEM career choice. | | | | |
| --- | --- | --- | --- | --- |
| Model | Variables | Log Odds Beta | SE | OR (95% CI) |
| One  Unadjusted  N = 855  Nagelkerke R^2^ = 0.15 | MA | -0.27* | 0.10 | 0.78 (0.64, 0.96) |
|  | MSE | 0.22 | 0.13 | 1.25 (0.96, 1.62) |
|  | MI | 0.58*** | 0.10 | 1.75(1.44, 2.12) |
| Two  GCSE adjusted  N = 808  Nagelkerke R^2^ = 0.20 | MA | -0.21 | 0.11 | 0.81 (0.65, 1.01) |
|  | MSE | -0.10 | 0.15 | 0.90 (0.67, 1.22) |
|  | MI | 0.47*** | 0.11 | 1.60(1.30, 1.97) |
| Three  SES + GCSE adjusted  N = 783  Nagelkerke R^2^ = 0.21 | MA | -0.24 | 0.11 | 0.79 (0.62, 0.98) |
|  | MSE | -0.10 | 0.16 | 0.90 (0.66, 1.23) |
|  | MI | 0.50*** | 0.11 | 1.65 (1.33, 2.04) |
| Notes: one twin out of each pair was selected to control for non-independence of observation  MA – maths anxiety, MSE – maths self-efficacy, MI – maths interest, GCSE – maths achievement (GCSE), SES – socioeconomic status.  * = p < .05 ** = p< .01, *** = p<.001. | | | | |

|  | | Supplementary Table 8: Zero-order correlations between all variables in sex-stratified samples *(N; 95% CIs)* | | | | | | |
| --- | --- | --- | --- | --- | --- | --- | --- | --- |
|  | SES | | MA | MA (independent of GA) | GA | MSE | MI | GCSE |
| Female |  | |  |  |  |  |  |  |
| MA | -0.01  (896; -0.07, 0.06) | | --- | --- | --- | --- | --- | --- |
| MA (independent of GA) | -0.01  (896; -0.07, 0.06) | | 0.94***  (946; -0.93, 0.94) | --- | --- | --- | --- | --- |
| GA | -0.01  (896; -0.08, 0.05) | | 0.35***  (946; 0.29, 0.40) | 0.00  (946; -0.06, 0.06) | --- | --- | --- | --- |
| MSE | 0.24***  (1393; 0.19, 0.28) | | -0.43***  (937; -0.48, -0.37) | -0.42***  (937; -0.46, -0.36) | -0.12***  (937; -0.18, -0.06) | --- | --- | --- |
| MI | 0.09***  (1393; 0.04, 0.14) | | -0.43***  (937; -0.48, -0.37) | -0.43***  (937; -0.48, -0.37) | -0.07  (937; -0.13, -0.01) | 0.54***  (1483, 0.50, 0.57) | --- | --- |
| GCSE | 0.44***  (3162; 0.41, 0.46) | | -0.28***  (866; -0.35, -0.22) | -0.26***  (866; -0.32, 0.20) | -0.13***  (866; -0.20, -0.07) | 0.68***  (1299; 0.65, 0.71) | 0.42***  (1299; 0.38,0.46) | --- |
| STEM career choice | 0.15***  (1386; 0.10, 0.20) | | -0.21***  (575; -0.28, 0.13) | -0.19***  (575; -0.27, -0.11) | -0.08  (575; -0.16, 0.01) | 0.24***  (730; 0.17, 0.31) | 0.27***  (730; 0.20, 0.33) | 0.30***  (1224; 0.25, 0.35) |
| Male |  | |  |  |  |  |  |  |
| MA | -0.06  (518; -0.14, 0.03) | | --- | --- | --- | --- | --- | --- |
| MA (independent of GA) | -0.04  (518; -0.13, 0.04) | | 0.95***  (539; 0.94, 0.95) | --- | --- | --- | --- | --- |
| GA | -0.04  (518; -0.14, 0.04) | | 0.32***  (539; 0.25, 0.40) | 0.00  (539; -0.08, 0.08) | --- | --- | --- | --- |
| MSE | 0.27***  (1026; -0.22, 0.33) | | -0.37***  (534; -0.44, -0.30) | -0.32***  (534; -0.39, -0.24) | -0.22***  (534; -0.30, -0.14) | --- | --- | --- |
| MI | 0.08**  (1027; 0.02,0.14) | | -0.39***  (535; -0.46, -0.32) | -0.39***  (535; -0.45, -0.31) | -0.09*  (535; 0.18, -0.01) | 0.50***  (1071; 0.45, 0.54) | --- | --- |
| GCSE | 0.40***  (2913; 0.37, 0.43) | | -0.31***  (486; -0.38, -0.23) | -0.28***  (496; -0.36, -0.20) | -0.12***  (496; -0.21, -0.03) | 0.60***  (936; 0.56, 0.64) | 0.40***  (936; 0.34, 0.44) | --- |
| STEM career choice | 0.05  (783; -0.02, 0.11) | | -0.32***  (280; -0.42, -0.21) | 0.30***  (280; -0.40, -0.18) | -0.12  (280; -0.22, 0.00) | 0.16**  (390; 0.06, 0.25) | 0.30***  (390; 0.21, 0.39) | 0.24***  (711; 0.17, 0.30) |
|  | | Notes: one twin out of each pair was selected to control for non-independence of observation  MA – maths anxiety, MSE – maths self-efficacy, MI – maths interest, GA – general anxiety, GCSE – maths achievement (GCSE), SES – socioeconomic status.  * = p < .05 ** = p< .01, *** = p<.001. | | | | | | |

| Supplementary Table 9: Variance inflation factors (VIFs) for predictor variables in each of the four binary logistic regression models used in the female sample. | | | |
| --- | --- | --- | --- |
| Predictor variables | Model one | Model two | Model three |
| Maths anxiety | 1.33 | 1.36 | 1.36 |
| Maths self-efficacy | 1.31 | 1.54 | 1.53 |
| Maths interest | 1.28 | 1.30 | 1.30 |
| Maths achievement (GCSE) | - | 1.35 | 1.49 |
| Socioeconomic status (SES) | - | - | 1.13 |

| Supplementary Table 10: Variance inflation factors (VIFs) for predictor variables in each of the four binary logistic regression models used in the male sample. | | | |
| --- | --- | --- | --- |
| Predictor variables | Model one | Model two | Model three |
| Maths anxiety | 1.30 | 1.35 | 1.43 |
| Maths self-efficacy | 1.28 | 1.53 | 1.58 |
| Maths interest | 1.30 | 1.31 | 1.32 |
| Maths achievement (GCSE) | - | 1.26 | 1.43 |
| Socioeconomic status (SES) | - | - | 1.22 |

| Supplementary Table 11: Binary logistic regression models assessing the joint influence of maths anxiety and maths motivation on STEM career choice in sex-stratified samples. | | | | | | | |
| --- | --- | --- | --- | --- | --- | --- | --- |
|  |  | Female sample  (N = 573;  Nagelkerke R^2^ = 0.16-23) | | | Male sample  (N = 277;  Nagelkerke R^2^ = 0.18-19) | | |
| Model | Variables | Log Odds Beta | SE | OR (95% CI) | Log Odds Beta | SE | OR (95%CI) |
| One  Unadjusted | MA | -0.13 | 0.14 | 0.88 (0.67, 1.16) | -0.47** | 0.18 | 0.62 (0.42, 0.89) |
|  | MSE | 0.57** | 0.19 | 1.77 (1.20, 2.60) | -0.01 | 0.21 | 0.98 (0.65, 1.50) |
|  | MI | 0.57*** | 0.14 | 1.77 (1.34, 2.33) | 0.63*** | 0.16 | 1.88 (1.37, 2.59) |
| Two  GCSE adjusted | MA | -0.09 | 0.15 | 0.92 (0.68, 1.24) | -0.46* | 0.19 | 0.63 (0.44, 0.91) |
|  | MSE | 0.23 | 0.22 | 1.26 (0.81, 1.96) | -0.28 | 0.25 | 0.75 (0.46, 1.22) |
|  | MI | 0.49** | 0.15 | 1.63 (1.21, 2.18) | 0.53** | 0.22 | 1.71 (1.24, 2.38) |
| Three  SES + GCSE adjusted | MA | -0.13 | 0.16 | 0.87 (0.64, 1.18) | -0.44* | 0.19 | 0.64 (0.44, 0.94) |
|  | MSE | 0.25 | 0.22 | 1.28 (0.82, 2.02) | -0.31 | 0.25 | 0.73 (0.44, 1.19) |
|  | MI | 0.52*** | 0.15 | 1.68 (1.25, 2.28) | 0.57*** | 0.17 | 1.78 (1.27, 2.49) |
| Note: MA – maths anxiety, MSE – maths self-efficacy, MI – maths interest, GCSE – maths achievement (GCSE), SES – socioeconomic status.  * = p < .05 ** = p< .01, *** = p<.001. | | | | | | | |
|  | | | | | | | |

| Supplementary Table 12: T-test results for the robustness checks on the socioeconomic status in participating compared to non- participating individuals. | | | | | |
| --- | --- | --- | --- | --- | --- |
|  | Data Present - Mean SES, N (% total sample) | | Data Missing - Mean SES, N (% total sample) | *T* | d |
| Maths anxiety | 0.27 N = 1489 (18.8%) | 0.13, N = 6419 (81.2%) | | -4.78*** | 0.14 |
| General anxiety | 0.27, N = 1489 (18.8%) | 0.13, N = 6419 (81.2%) | | -4.78*** | 0.14 |
| Mathematics self-efficacy | 0.20, N = 2258 (28.6%) | 0.13, N = 5350 (67.7%) | | -2.68** | 0.07 |
| Mathematics interest | 0.20, N = 2258 (28.6%) | 0.13, N = 5350 (67.7%) | | -2.68** | 0.07 |
| Maths achievement (GCSE) | 0.23, N = 6409 (81.0%) | -0.20, N = 1499 (19.0%) | | -14.16*** | 0.44 |
| STEM career choice | 0.45, N = 2254 (28.5%) | 0.03, N = 5654 (71.5%) | | -17.24*** | 0.43 |
| Notes: one twin out of each pair was selected to control for non-independence of observation; SES is a standardised variable.  * = p < .05 ** = p< .01, *** = p<.001. | | | | | |
